# β_2_-Microglobulin Amyloid Fibrils Are Nanoparticles That Disrupt Lysosomal Membrane Protein Trafficking and Inhibit Protein Degradation by Lysosomes*

**DOI:** 10.1074/jbc.M114.586222

**Published:** 2014-11-05

**Authors:** Toral Jakhria, Andrew L. Hellewell, Morwenna Y. Porter, Matthew P. Jackson, Kevin W. Tipping, Wei-Feng Xue, Sheena E. Radford, Eric W. Hewitt

**Affiliations:** From the School of Molecular and Cellular Biology and Astbury Centre for Structural Molecular Biology, University of Leeds, Leeds LS2 9JT, United Kingdom

**Keywords:** Amyloid, Fibril, Lysosome, Membrane Trafficking, Protein Degradation

## Abstract

Fragmentation of amyloid fibrils produces fibrils that are reduced in length but have an otherwise unchanged molecular architecture. The resultant nanoscale fibril particles inhibit the cellular reduction of the tetrazolium dye 3-(4,5-dimethylthiazol-2-yl)-2,5-diphenyltetrazolium bromide (MTT), a substrate commonly used to measure cell viability, to a greater extent than unfragmented fibrils. Here we show that the internalization of β_2_-microglobulin (β_2_m) amyloid fibrils is dependent on fibril length, with fragmented fibrils being more efficiently internalized by cells. Correspondingly, inhibiting the internalization of fragmented β_2_m fibrils rescued cellular MTT reduction. Incubation of cells with fragmented β_2_m fibrils did not, however, cause cell death. Instead, fragmented β_2_m fibrils accumulate in lysosomes, alter the trafficking of lysosomal membrane proteins, and inhibit the degradation of a model protein substrate by lysosomes. These findings suggest that nanoscale fibrils formed early during amyloid assembly reactions or by the fragmentation of longer fibrils could play a role in amyloid disease by disrupting protein degradation by lysosomes and trafficking in the endolysosomal pathway.

## Teaser

**Background:** The causative agents and pathological mechanisms of amyloid disease are poorly understood.

**Results:** β_2_-Microglobulin amyloid fibrils display length-dependent internalization, alter trafficking of lysosomal membrane proteins, and inhibit the degradation of proteins by lysosomes.

**Conclusion:** Amyloid fibrils act as nanoparticles that disrupt membrane trafficking and protein degradation.

**Significance:** Fibril length, by determining access to intracellular compartments, may contribute to amyloid disease.

## Introduction

The amyloidoses are a class of human disorders that include the neurological conditions Alzheimer, Parkinson, Huntington, and Creutzfeldt-Jakob diseases; the metabolic disorder type II diabetes mellitus; and the systemic condition dialysis-related amyloidosis (1–3). Irrespective of the sequence and native structure of the culprit protein, amyloid fibrils share a common characteristic cross-β architecture (1, 2). In many amyloid diseases, fibril formation is associated with cellular dysfunction and tissue destruction, although the molecular and cellular mechanisms of amyloid disease remain unclear (2, 4, 5). It is imperative, therefore, to characterize species associated with amyloid formation and to elucidate how these species interact with cells and affect cellular function.

Numerous studies have shown that oligomeric intermediates of amyloid assembly can be cytotoxic *in vitro* and *in vivo* (6–11) and impair memory and long-term potentiation (12–14). By contrast with oligomeric species, amyloid fibrils have been shown to exhibit limited, if any, cytotoxicity (6–11) and it has even been suggested that amyloid fibrils represent inert end products of amyloid assembly. In other experiments, however, some fibril samples have been shown to be cytotoxic (15–18), to possess cytotoxic potential that is dependent on the nature of the preparation (19–24), and, upon depolymerization, to act as a source of cytotoxic oligomers and protofilaments (25–27).

Further evidence for a potential role for fibrils in amyloid disease mechanisms was provided by our observations that amyloid fibrils formed *in vitro* from β_2_-microglobulin (β_2_m),^5^ α-synuclein and hen egg white lysozyme disrupt artificial lipid membranes (28, 29). Intriguingly, nanoscale fibrils produced by fragmentation disrupted these lipid membranes to a greater extent than their longer unfragmented precursors (28), potentially via the interaction of fibril ends with lipid bilayers (30). Fragmented fibrils also inhibit the cellular reduction of the tetrazolium dye 3-(4,5-dimethylthiazol-2-yl)-2,5-diphenyltetrazolium bromide (MTT) (28), a substrate commonly used to assay cell viability (31). However, the precise nature of this cellular perturbation and why it is dependent on fibril length remained unclear.

In this study, the amyloidogenic protein β_2_m, the causative agent of dialysis-related amyloidosis (32), was used to investigate the cellular effects of fragmented amyloid fibrils. Despite inhibiting the cellular reduction of MTT, we show that fragmented β_2_m fibrils have no effect on cell viability. β_2_m fibrils instead exhibited length-dependent uptake by cells, with fragmented fibrils (nanometer length) being internalized and sorted to lysosomes to a greater extent than longer (micrometer length) unfragmented fibrils. Moreover, fragmented β_2_m fibrils altered the trafficking of the lysosomal membrane proteins LAMP-1 and CD63 and inhibited the degradation of ovalbumin, a model protein substrate for lysosomal proteases. These results reveal lysosomes as a cellular target for amyloid fibrils and suggest that nanoscale-length fibrils or species derived from these fibrils may contribute to amyloid disease.

## EXPERIMENTAL PROCEDURES

### 

#### 

##### Preparation of Fibril Samples

Fibrils of human β_2_m were formed from recombinant protein as described previously at a monomer-equivalent concentration of 120 μm (28). Labeling of β_2_m with tetramethylrhodamine (TMR, Molecular Probes) was performed as described previously (33). TMR-labeled fibrils were formed from a 1:9 mixture of TMR-labeled:unlabeled β_2_m. Fragmented fibrils were prepared by fragmenting samples of unfragmented fibrils by stirring at 1000 rpm at 25 °C for 2 days using a custom-made precision stirrer (built by the workshop of the School of Physics and Astronomy, University of Leeds, UK) (28). β_2_m fibril samples were imaged using atomic force microscopy, and fibril length distributions were analyzed as described previously (34).

##### Cell Culture

The SH-SY5Y neuroblastoma cell line was cultured as described previously (28). Cells were incubated with either 1.2 or 6.0 μm (monomer-equivalent concentration) of β_2_m fibril samples or β_2_m monomers for up to 48 h.

##### Analysis of Cell Viability

SH-SY5Y cells were cultured in 96-well plates (20,000 cells/well for MTT and WST-1 assays and 10,000 cells/well for the ATP assay) for 24 h in 200 μl of growth medium. The medium was then replaced, and β_2_m fibril samples or controls (fibril growth buffer or 0.1% (w/v) NaN_3_) were incubated with the cells for a further 24 h. Each experiment consisted typically of two to three independent experiments, each containing five replicates per condition. For inhibition of fibril internalization, 5 μm Dynasore (Merck) was incubated with the cells for 30 min prior to addition of fibril samples. The MTT assay was performed as described previously (28). To assay for WST-1 metabolism, 100 μl of medium was removed from each well, and 10 μl of WST-1 cell proliferation reagent (Roche) was added. Cells were then incubated for 1 h, and absorbance was read directly at 450 nm using a Powerwave XS2 plate reader (BioTek). For the MTT and WST-1 assays, the results were normalized using the signal for cells incubated with the fibril growth buffer as 100% viability, and cells were treated with NaN_3_ as 0% viability. Cellular ATP was measured with the ATPLite Luminescence ATP detection assay system (PerkinElmer Life Sciences) according to the protocol of the manufacturer, and luminescence was measured on a POLARstar OPTIMA plate reader (BMG Labtech). ATP concentrations were calculated for the volume of cell culture medium.

##### Propidium Iodide Staining

5 × 10^5^ SH-SY5Y cells were cultured in 12-well plates for 24 h and incubated with β_2_m fibril samples for 24 h. As a positive control, cells were incubated with 600 mm H_2_O_2_ for 5 h. Cells were detached from the culture plates and incubated with 75 μm propidium iodide prior to analysis on a BD-LSRFortessa flow cytometer (BD Biosciences). 10,000 gated events were recorded for three independent experiments, each containing three replicates per condition. Propidium iodide-positive cells were defined as those that stained with the dye to the same level as cells incubated with H_2_O_2_.

##### Analysis of Cell-associated β_2_m Monomer and Fibrils

SH-SY5Y cells were incubated with TMR-β_2_m-labeled fibrils or TMR-β_2_m monomers (9:1 ratio unlabeled:TMR-labeled protein) for 4 h. Prior to live cell imaging, cells were incubated with 100 nm LysoTracker Green (Molecular Probes) for 30 min. To inhibit endocytic pathways, cells were incubated with 5 μm Dynasore for 30 min prior to incubation with fibril samples. Imaging was performed using a Zeiss LSM510 META laser scanning confocal microscope and an inverted AxioVert 200 M microscope with a ×40 objective. The total cell-associated TMR-β_2_m fluorescence for 10,000 gated events was quantified with a FACSCalibur flow cytometer (BD Biosciences).

##### NIAD-4 Staining of β_2_m Amyloid Fibrils

To measure NIAD-4 (ICX Nomadics) binding to β_2_m monomer and fragmented fibrils *in vitro* at pH values of 7.4 and 4.5, samples containing 12 μm (monomer-equivalent concentration) of β_2_m fibril samples or monomers were incubated with 7.5 μm NIAD-4 for 1 h at 25 °C. The fluorescence emission spectra of the samples (excitation wavelength of 500 nm) were subsequently collected after incubation for 1 h at 25 °C using a PTI Quantamaster fluorescence spectrometer. To detect cell-associated amyloid fibrils, SH-SY5Y cells were incubated with β_2_m samples for 16 h prior to staining with 100 nm NIAD-4 and either 100 nm LysoTracker Deep Red (Molecular Probes) or 0.5 μg/ml CellMask Deep Red plasma membrane stain (Molecular Probes). Imaging was performed using a Zeiss LSM700 confocal microscope with a ×63 objective.

##### Analysis of Lysosome Membrane Permeability

To analyze lysosome integrity using live cell confocal microscopy and flow cytometry, cells were stained with 100 nm LysoTracker Green 30 min prior to analysis. As a positive control for lysosome membrane permeabilization, cells were incubated for 15 min with either 6 or 15 μm sphingosine. Cells were imaged using a Zeiss LSM700 confocal microscope with a ×63 objective. Flow cytometry was performed using a BD-LSRFortessa flow cytometer, with 10,000 gated events recorded for three independent experiments, each containing three replicates per condition. For subcellular fractionation, 2 × 10^7^ cells were resuspended into 1 ml of homgenization buffer (10 mm acetic acid, 1 mm EDTA, 190 mm sucrose, and 10 mm triethanolamine (pH 7.4)), homogenized with a ball bearing homogenizer (Isobiotec) as described previously (35), and centrifuged at 400 × *g* for 10 min in a microcentrifuge to generate a postnuclear supernatant. This was centrifuged at 100,000 × *g* for 1 h (S100-AT3 rotor, Sorvall). The activity of α-*N*-acetylgalactosaminidase in the resultant pellet and supernatant fractions was assayed as described previously (36).

##### Analysis of the Expression and Localization of LAMP-1 and CD63

To visualize the cellular distribution of LAMP-1 and CD63 cells by immunofluorescence microscopy, cells were fixed and permeabilized as described previously (35) before staining with rabbit anti-LAMP1 (Sigma-Aldrich) and mouse anti-CD63 (clone MEM-259, AbD Serotec) antibodies. CD63 staining was detected with an anti-rabbit FITC antibody, and LAMP1 staining was detected with an anti-mouse Texas Red antibody (BD Biosciences). Cells were imaged using a Zeiss LSM700 confocal microscope with a ×63 objective. Cell surface expression of LAMP-1 and CD63 cells was analyzed by flow cytometry. To inhibit protein synthesis, cells were preincubated for 1 h with 100 μg/ml cycloheximide prior to the addition of β_2_m samples. Cells were then resuspended in PBS, 0.2% BSA, and 10% Mouse Seroblock FcR (AbD Serotec) and incubated for 30 min on ice. To detect cell surface expression of LAMP-1 and CD63, cells were stained with PE-Cy5 conjugated mouse anti-human LAMP-1 (clone H4A3, BD Biosciences) and FITC-conjugated mouse anti-human CD63 (clone MEM-259, Genetex), respectively. To measure nonspecific background antibody binding, controls were included in which cells were stained with the PE-Cy5-conjugated mouse IgG_1_ κ (BD Biosciences) and FITC-conjugated mouse IgG_1_ (Genetex) isotype controls, respectively, for the LAMP-1 and CD63 antibodies. Cell-associated antibody fluorescence was quantified by flow cytometry with a BD-LSRFortessa, and 10,000 gated events were recorded for three independent experiments, each containing three replicates per condition. For each experimental condition, the geometric mean fluorescence for cells stained with the isotype controls was subtracted from the geometric mean fluorescence for cells stained with the LAMP-1 and CD63 antibodies. The resultant fluorescence value was then normalized to that of cells incubated for 0 h with β_2_m samples. The total cellular levels of LAMP-1 and CD63 were determined by immunoblotting of cell lysates with mouse anti-human LAMP-1 (clone H4A3, Santa Cruz Biotechnology) and rabbit anti-human CD63 (clone MX-49.129.5, Santa Cruz Biotechnology) antibodies, respectively. Immunoblots were also probed with a GAPDH antibody (clone 6C5, Abcam) as a loading control.

##### Analysis of the Lysosomal Degradation of Ovalbumin

After incubation with β_2_m samples for 24 h, cells were washed and incubated for 6 h with 15 μg/ml ovalbumin, which corresponded to a 2:3 mixture of ovalbumin-Alexa Fluor 647 (Ova-647, Molecular Probes):unlabeled ovalbumin. Cells were then washed to remove non-cell-associated ovalbumin and either imaged or incubated for a further 24 h before imaging. Prior to imaging, lysosomes were stained with 100 nm LysoTracker Green and viewed with a Zeiss LSM700 confocal microscope with a ×63 objective. Total cell-associated Ova647 fluorescence was quantified by flow cytometry with a BD-LSRFortessa (BD Biosciences), and 10,000 gated events were recorded for three independent experiments, each containing three replicates per condition.

##### Analysis of the Activity of Lysosomal β-Glucocerebrosidase and β-Galactosidase

After incubation with β_2_m samples for 24 h, cells were washed and incubated with either 58 μm 5-(pentafluorobenzoylamino)fluorescein di-β-d-glucopyranoside (Invitrogen) or 33 μm 5-dodecanoylaminofluorescein di-β-d-galactopyranoside (C_12_FDG, Invitrogen) for 1 h at 37 °C. β-glucocerebrosidase cleaves the non-fluorescent substrate 5-(pentafluorobenzoylamino)fluorescein di-β-d-glucopyranoside to yield the green-fluorescent 5-(pentafluorobenzoylamino)fluorescein dye, whereas β-galactosidase cleaves the non-fluorescent substrate C_12_FDG to yield fluorescent fluorescein that can be detected by flow cytometry (37, 38). As a control, cells were preincubated for 1 h with 1 μm conduritol B epoxide (Sigma-Aldrich) and 1 mm phenylethyl β-d-thiogalactopyranoside (Invitrogen) to inhibit the activities of β-glucocerebrosidase and β-galactosidase, respectively (39, 40). Cell-associated fluorescence was quantified with a BD-LSRFortessa flow cytometer, and 10,000 gated events were recorded for two independent experiments, each containing three replicates per condition.

##### Statistical Analysis

*p* values were determined using two-tailed independent two-sample Student's *t* test between sample pairs.

## RESULTS

### 

#### 

##### Fragmented β_2_m Fibrils Inhibit Cellular MTT Reduction but Do Not Cause Cell Death

**FIGURE 1.**
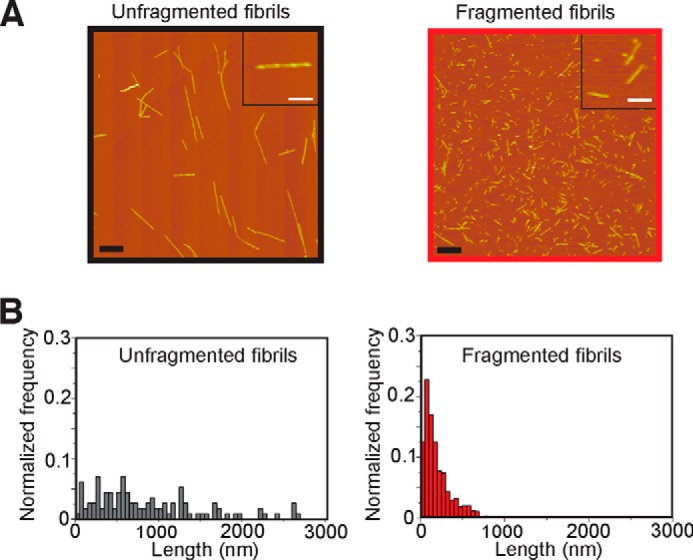
**Atomic force microscopy characterization of β_2_m fibrils.** *A*, atomic force microscopy images of unfragmented and fragmented β_2_m fibrils. *Scale bars* = 1 μm (*main images*) and 250 nm (*insets*). *B*, fibril length distributions. The average fibril length was calculated from >200 particles to be 1.30 ± 0.05 μm and 0.30 ± 0.01 μm for the unfragmented and fragmented fibrils, respectively.

Incubation of β_2_m at pH 2.0 under quiescent conditions results in the formation of straight and twisted amyloid-like fibrils (Fig. 1*A*) that closely resemble those present in *ex vivo* amyloid deposits (32, 41–43). These fibrils were then fragmented with a precision stirrer (Fig. 1*A*) (28), reducing the average fibril length from ∼1.3 μm to ∼300 nm (Fig. 1*B*). Fragmentation of β_2_m fibrils does not generate species that are detected by the oligomer-specific antibody A11 (6, 28), but they retain their structural integrity, as judged by Fourier transform infrared spectroscopy and the ability to bind the amyloid-specific antibody WO1 (28). All subsequent experiments were performed with fibril samples of these lengths.

**FIGURE 2.**
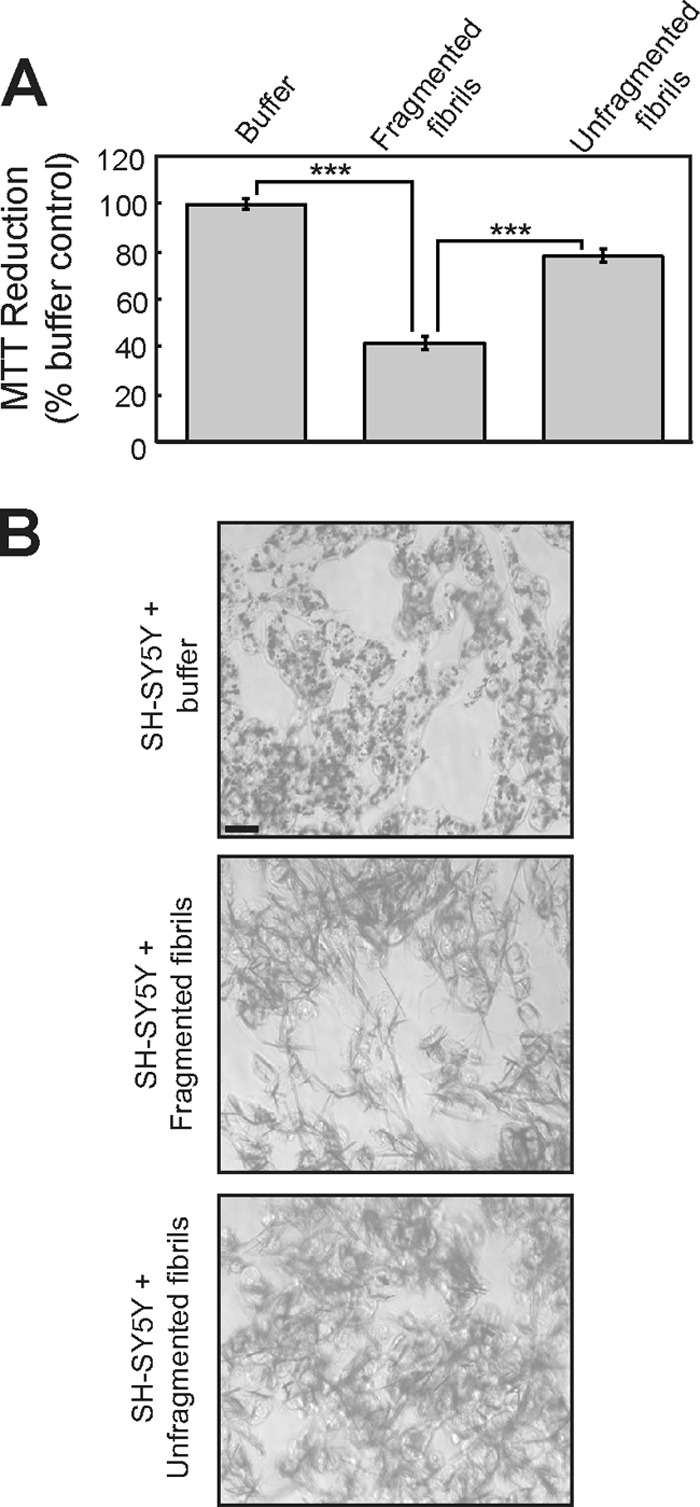
**Fragmented fibrils of β_2_m inhibit the cellular reduction of MTT.** *A*, SH-SY5Y cells were incubated for 24 h with 1.2 μm (monomer-equivalent concentration) fragmented or unfragmented β_2_m fibrils, and the reduction of MTT was assayed. The percentage of MTT reduction relative to control cells incubated with the fibril growth buffer was plotted. The *error bars* represent mean ± 1 S.E. over a total of 15 replicates. ***, *p* < 0.001. *B*, cells incubated with MTT were imaged by phase-contrast microscopy to visualize the reduced MTT formazan (*dark staining*). *Scale bar* = 20 μm.

The effect of the fibrils on the neuroblastoma cell line SH-SY5Y was then analyzed using an MTT assay. In this procedure, cellular reduction of the tetrazolium dye MTT is measured, and this assay can be used as an indicator of cell viability (31). As reported previously (28), fragmented β_2_m fibrils inhibited the reduction of MTT to a greater extent than either unfragmented fibrils or fibril growth buffer (Fig. 2*A*). Studies examining the cellular mechanism of MTT reduction have shown that MTT is reduced to produce an insoluble colored formazan salt that localizes to intracellular granule-like structures before being exocytosed to the cell surface, where it forms needle-like crystals (31, 44). Other amyloidogenic sequences, Aβ_1–40_ and amylin, greatly increase the proportion of reduced MTT formazan present in these extracellular needle-like crystals by increasing the exocytosis of MTT formazan (45–47). Analysis of SH-SY5Y cells revealed that MTT formazan was present in intracellular punctate structures in cells incubated with fibril growth buffer, whereas cells incubated with β_2_m fibrils exhibited a marked increase in extracellular needle-like crystals of reduced MTT formazan (Fig. 2*B*). These data suggest that the inhibition of MTT reduction by fragmented β_2_m fibrils may result from increased secretion of the MTT reduction product, which, in turn, may inhibit cellular uptake of MTT (45–47).

**FIGURE 3.**
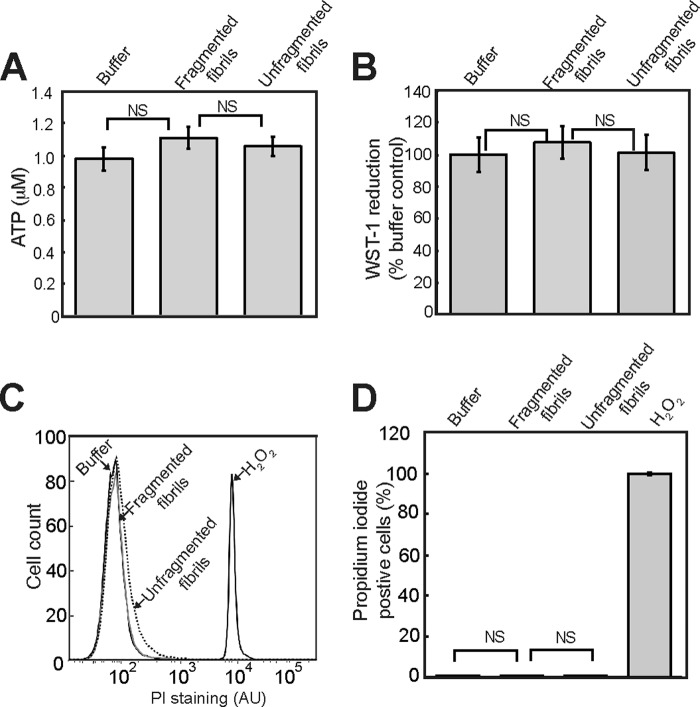
**Fragmented fibrils of β_2_m do not reduce cell viability.** *A* and *B*, SH-SY5Y cells were incubated for 24 h with 1.2 μm (monomer-equivalent concentration) fragmented or unfragmented β_2_m fibrils, and the cellular level of ATP (*A*) and the reduction of WST-1 were assayed (*B*). The ATP concentration and the WST-1 signal relative to control cells incubated with the fibril growth buffer were plotted. The *error bars* represent mean ± S.E. over a total of 15 (ATP) and 10 (WST-1) replicates. *NS*, *p* ≥ 0.05. SH-SY5Y cells were stained with propidium iodide (*PI*) after 24 h incubation with either β_2_m fragmented or unfragmented fibrils and analyzed by flow cytometry. H_2_O_2_, which increases plasma membrane permeability, was used as positive control. *C*, representative flow cytometry profiles. *AU*, arbitrary units. *D*, the percentage of propidium iodide-stained cells was plotted. The *error bars* represent mean ± 1 S.E. over a total of nine replicates.

To determine whether the inhibition of cellular MTT reduction by fragmented β_2_m fibrils corresponds to a loss of cell viability, cellular ATP levels were assayed. No pronounced decrease in cellular ATP levels was observed in SH-SY5Y cells incubated with either fragmented or unfragmented fibrils (Fig. 3*A*). The effect of fibrils on reduction of the tetrazolium salt WST-1 was also assayed. In contrast to MTT, which is reduced intracellularly, WST-1 reduction is thought to occur at the plasma membrane (31, 44). Fragmented β_2_m fibrils had no significant effect on the reduction of WST-1 by SH-SY5Y cells (Fig. 3*B*), suggesting that the overall reductive capacity of the cells is not diminished. Staining of cells with the plasma membrane-impermeable dye propidium iodide is indicative of cells that have undergone necrosis or late-stage apoptosis (48). No significant increase in the percentage of cells stained with propidium iodide was observed when cells were incubated with fragmented β_2_m fibrils for 24 h (Fig. 3, *C* and *D*), indicating that the plasma membrane is intact in fibril-treated cells. Taken together, these data demonstrate that fragmented β_2_m fibrils do not have a significant effect on cell viability. Nonetheless, the inhibition of MTT reduction suggests that β_2_m fibrils do have a length-dependent effect on some facet of cellular physiology.

##### β_2_m Fibrils Exhibit Length-dependent Internalization, and the Inhibition of Fibril Uptake Rescues the Cellular Reduction of MTT

**FIGURE 4.**
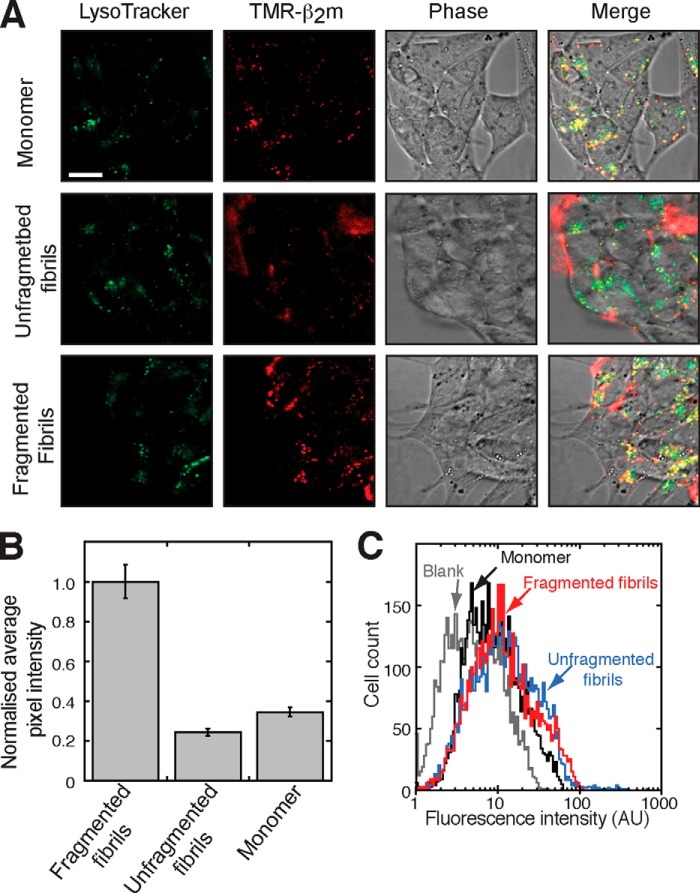
**Fragmented fibrils of β_2_m are internalized and accumulate in lysosomes.** *A*, SH-SY5Y cells were incubated with 1.2 μm (monomer-equivalent concentration) TMR-labeled fragmented or unfragmented β_2_m fibrils or 1.2 μm TMR-labeled β_2_m monomers for 4 h. Labeled samples contained 10% TMR-labeled β_2_m and 90% unlabeled monomer. Prior to imaging, the lysosomes were stained with the lysotrophic dye LysoTracker Green. Cell-associated fluorescence was visualized by live cell confocal microscopy. TMR, Lysotracker Green, and phase-contrast images are shown individually and merged. In the *merged images*, *yellow* is indicative of colocalization of the TMR-labeled β_2_m and the LysoTracker Green dye. *Scale bar* = 10 μm. *B*, the mean pixel intensity of intracellular TMR-labeled β_2_m within SH-SY5Y cells was quantified from the confocal microscopy images for 60 cells. The data were normalized to the pixel count of samples incubated with fragmented fibrils. The *error bars* represent mean ± S.E. *C*, total cell-associated TMR fluorescence was quantified by flow cytometry. *AU*, arbitrary units.

We have shown previously that β_2_m fibrils are internalized and trafficked to lysosomes and that they are resistant to lysosomal proteolysis (33, 49). We therefore investigated the relationship between fibril length, fibril trafficking to lysosomes, and the inhibition of MTT reduction. SH-SY5Y cells were incubated for 4 h with either TMR-labeled monomeric β_2_m or fibrils that incorporate TMR-labeled β_2_m, and the localization of the fluorescently labeled protein was visualized using live cell confocal microscopy. Consistent with the known uptake and degradation of monomeric β_2_m within lysosomes (33, 49), cells incubated with TMR-labeled monomeric β_2_m exhibited intracellular TMR fluorescence that colocalized in part with LysoTracker Green (Fig. 4*A*), a dye that accumulates in acidic compartments. Cells incubated with unfragmented fibrils had limited intracellular TMR-associated fluorescence. The fibrils were instead localized predominantly in close proximity to the extracellular side of the plasma membrane (Fig. 4*A*). Conversely, cells incubated with TMR-labeled fragmented β_2_m fibrils displayed more extensive intracellular punctate fluorescence that colocalized with LysoTracker Green (Fig. 4*A*). Quantification of intracellular TMR-β_2_m fluorescence revealed a >5-fold increase in intracellular β_2_m in cells incubated with fragmented fibril samples compared with cells incubated with unfragmented fibrils (Fig. 4*B*). By contrast, total cell-associated fluorescence, measured by flow cytometry, was comparable for cells incubated with either TMR-labeled fragmented or unfragmented fibrils (Fig. 4*C*).

**FIGURE 5.**
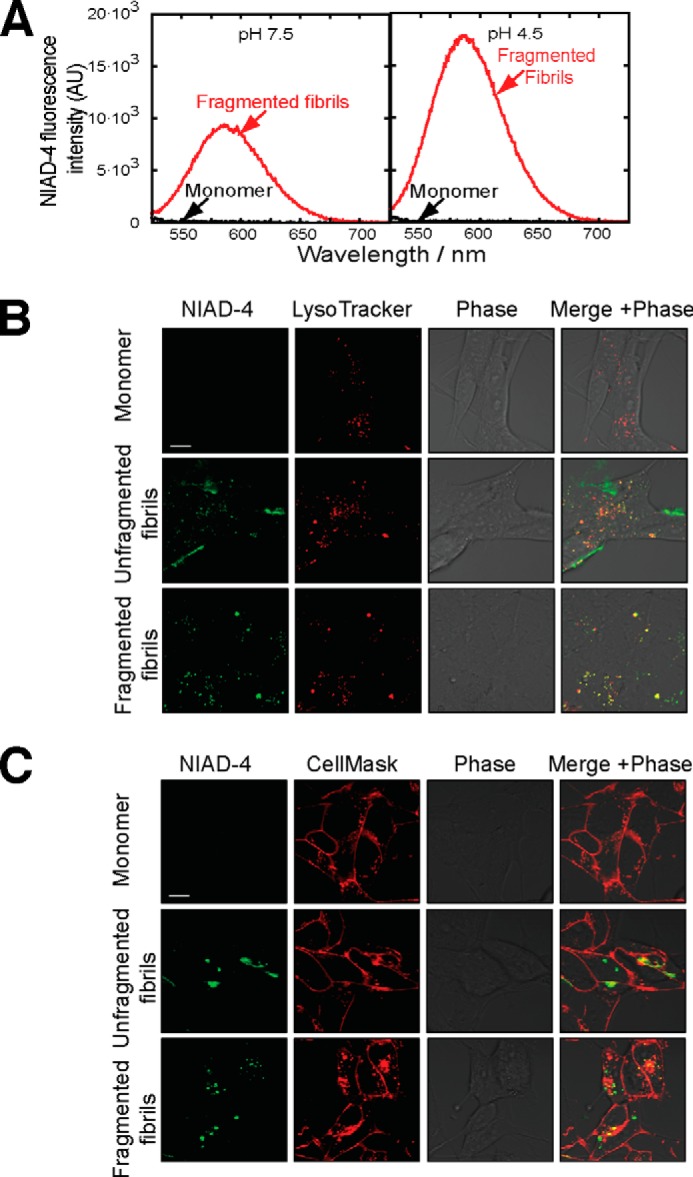
**Fragmented fibrils of β_2_m retain elements of amyloid structure after internalization.** *A*, fluorescence emission spectra of NIAD-4 in the presence of monomeric β_2_m or fragmented β_2_m fibrils at pH 7.5 and 4.5. *AU*, arbitrary units. *B* and *C*, SH-SY5Y cells were incubated for 16 h with 1.2 μm (monomer-equivalent concentration) fragmented or unfragmented β_2_m fibrils or β_2_m monomers. Prior to imaging, the cells were stained with the amyloid-specific dye NIAD-4 and with either LysoTracker Far Red (*B*) to label acidic compartments or CellMask Deep Red plasma membrane stain (*C*). Cell-associated fluorescence was visualized by live cell confocal microscopy. In the *merged images*, *yellow* indicates colocalization of NIAD-4 (*green*) with either LysoTracker Far Red or CellMask Deep Red. *Scale bar* = 10 μm.

To determine whether β_2_m fibrils retain an amyloid architecture after internalization, SH-SY5Y cells incubated with unlabeled β_2_m fibrils were stained with the amyloid-specific dye NIAD-4 (50). *In vitro* experiments showed that the fluorescence intensity of NIAD-4 increases upon binding to β_2_m fibrils at both pH 7.5 (the pH of culture medium and the cytosol) and pH 4.5 (lysosomal pH) but not when NIAD-4 is incubated with monomeric β_2_m at either pH (Fig. 5*A*). Likewise, no staining with NIAD-4 was observed for cells incubated with monomeric β_2_m (Fig. 5, *B* and *C*). Punctate NIAD-4 fluorescence was observed for cells incubated with fragmented β_2_m fibrils that colocalized with LysoTracker Far Red staining (Fig. 5*B*), consistent with their internalization and the resistance of β_2_m fibrils to digestion by lysosomal proteases (49). However, for cells incubated with unfragmented fibrils, NIAD-4 fluorescence was localized predominantly in proximity to the plasma membrane labeled with CellMask Far Red (Fig. 5*C*). These data demonstrate that, by reducing fibril length, fragmentation enables increased trafficking of β_2_m fibrils to lysosomes and that these cell-associated β_2_m fibrils retain, at least in part, a cross-β architecture.

**FIGURE 6.**
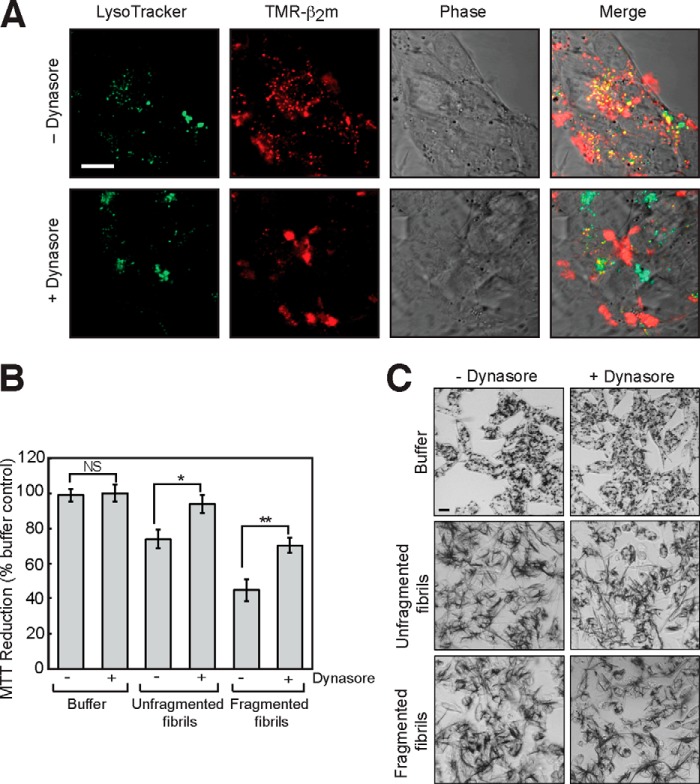
**The inhibition of MTT metabolism is dependent on the internalization of fragmented fibrils of β_2_m.** *A*, SH-SY5Y cells were incubated with 1.2 μm (monomer-equivalent concentration) TMR-labeled fragmented β_2_m fibrils for 24 h in the presence or absence of 5 μm Dynasore. Prior to imaging, acidic compartments were stained with LysoTracker Green. Cell-associated fluorescence was visualized by live cell confocal microscopy. In the *merged images*, *yellow* indicates colocalization of the TMR-labeled β_2_m (*red*) and the LysoTracker Green. *Scale bar* = 10 μm. *B*, the effect of Dynasore on the cellular reduction of MTT by cells incubated with fibril growth buffer or with either fragmented or unfragmented fibrils was assayed. The percentage of MTT reduction relative to control cells incubated with the fibril growth buffer in the absence of Dynasore was plotted. The *error bars* represent mean ± 1 S.E. over a total of 25 replicates. *NS*, *p* > 0.05; *, *p* < 0.05; **, *p* < 0.01. *C*, cells incubated with MTT were imaged by phase-contrast microscopy to visualize the reduced MTT formazan (*dark staining*). *Scale bar* = 20 μm.

The above results suggest a direct relationship between the level of β_2_m fibril internalization and the effect of fibrils on the cellular reduction of MTT. To confirm whether this is the case, the effect of inhibiting endocytic pathways on the reduction of MTT by fibril-treated cells was examined. SH-SY5Y cells were incubated with Dynasore, an inhibitor of dynamin-dependent endocytosis (51), and the effects on fibril uptake and MTT metabolism were monitored using confocal microscopy and the MTT assay, respectively. Dynasore inhibited the internalization of β_2_m fragmented fibrils by SH-SY5Y cells (Fig. 6*A*) and partially rescued the inhibition of MTT reduction by β_2_m fibrils (Fig. 6*B*). Correspondingly, Dynasore decreased, in part, the formation of extracellular needle-like crystals of reduced MTT formazan by cells incubated with β_2_m fibrils (Fig. 6*C*). Therefore, these data suggest that access to intracellular compartments via dynamin-dependent endocytosis is required for the β_2_m fibril-associated effects on cellular function measured in the MTT assay.

##### Fragmented β_2_m Fibrils Do Not Increase Lysosome Membrane Permeability

**FIGURE 7.**
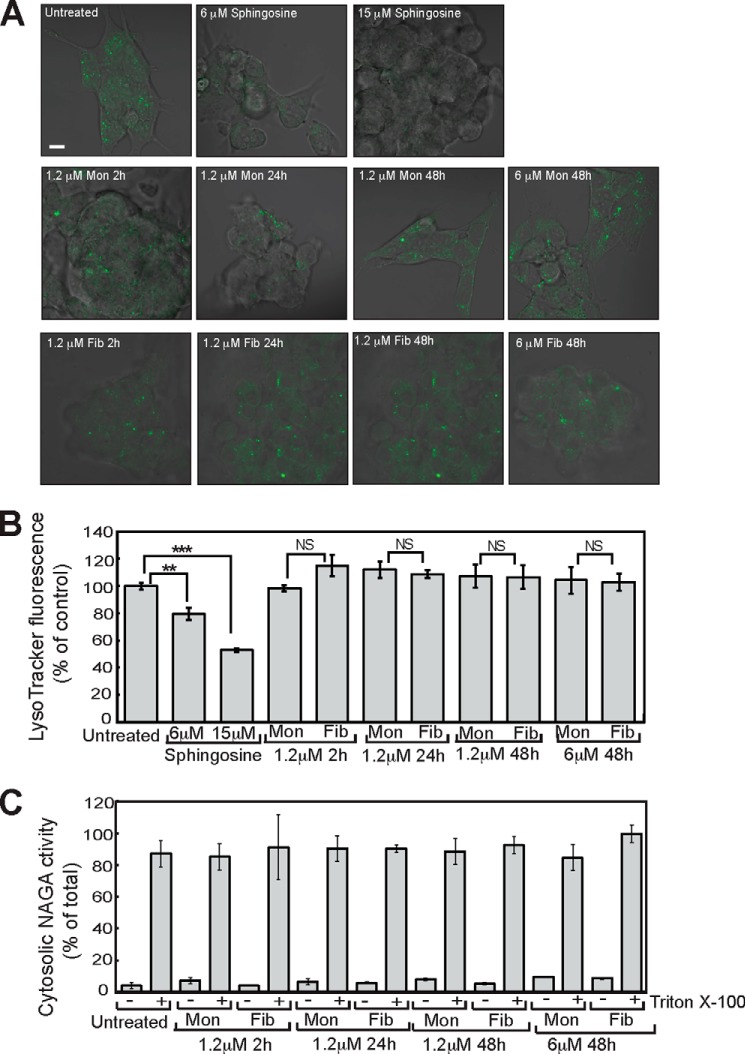
**Fragmented β_2_m fibrils do not increase lysosomal membrane permeability.** *A*, SH-SY5Y cells were incubated with fragmented β_2_m fibrils (*Fib*) or β_2_m monomers (*Mon*) for up to 48 h. The cells were stained with LysoTracker Green and visualized by live cell confocal microscopy, and cell-associated fluorescence was quantified by flow cytometry. Incubation with either 6 or 15 μm sphingosine for 30 min was used as a positive control for increased lysosomal membrane permeabilization. *Scale bar* = 10 μm. *B*, merged LysoTracker Green fluorescence and phase contrast images. LysoTracker Green fluorescence detected by flow cytometry was plotted as a percentage of that of cells incubated for 0 h in the presence of β_2_m samples. *Error bars* indicate mean ± S.E. over a total of nine replicates. *NS*, *p* ≥ 0.05; **, *p* < 0.01; ***, *p* < 0.001. *C*, postnuclear supernatants prepared from SH-SY5Y cells were centrifuged for 1 h at 100,000 × *g*. The activity of α-*N*-acetylgalactosaminidase in the cytosolic (supernatant) fractions was plotted as a percentage of the activity in the cytosolic and membrane (pellet) fractions combined. As a positive control for disruption of the lysosomal membrane, Triton X-100 was added to the postnuclear supernatants prior to centrifugation. *Error bars* indicate mean ± S.D. over a total of four replicates.

In previous work, we have shown that fragmented β_2_m fibrils interact with and disrupt artificial lipid membranes and that this is enhanced by endosomal lipids and an acidic pH (28–30, 52). We therefore examined whether the internalization of β_2_m fibrils results in an increase in the permeability of the lysosomal membrane. Sphingosine, a positive control, known to permeabilize the lysosomal membrane (53), caused a significant reduction in the cell-associated fluorescence of cells stained with LysoTracker Green when either visualized by live cell confocal microscopy or quantified by flow cytometry (Fig. 7, *A* and *B*). In contrast, fragmented β_2_m fibrils did not reduce the LysoTracker Green staining of cells (Fig. 7, *A* and *B*). Even when cells were incubated for 48 h with 6.0 μm (monomer-equivalent concentration) of fragmented β_2_m fibrils, no reduction in LysoTracker staining was detected (Fig. 7, *A* and *B*), therefore suggesting that fibrils do not increase permeability of the lysosomal membrane. Increased lysosome membrane permeability may also release soluble lysosomal hydrolases into the cytosol. SH-SY5Y cells were therefore incubated with fragmented β_2_m fibrils and fractionated by centrifugation into soluble (cytosol) and pellet (membrane) fractions. In both control cells and cells incubated with fragmented β_2_m fibrils, activity of the lysosomal hydrolase α-*N*-acetylgalactosaminidase was present predominantly in the membrane-fraction, with negligible activity in the cytosol fraction (Fig. 7*C*). Therefore, these experiments provide no evidence that fragmented β_2_m fibrils cause substantive damage to the lysosome membrane.

##### Fragmented β_2_m Fibrils Increase the Cell Surface Expression of Lysosomal Membrane Proteins

**FIGURE 8.**
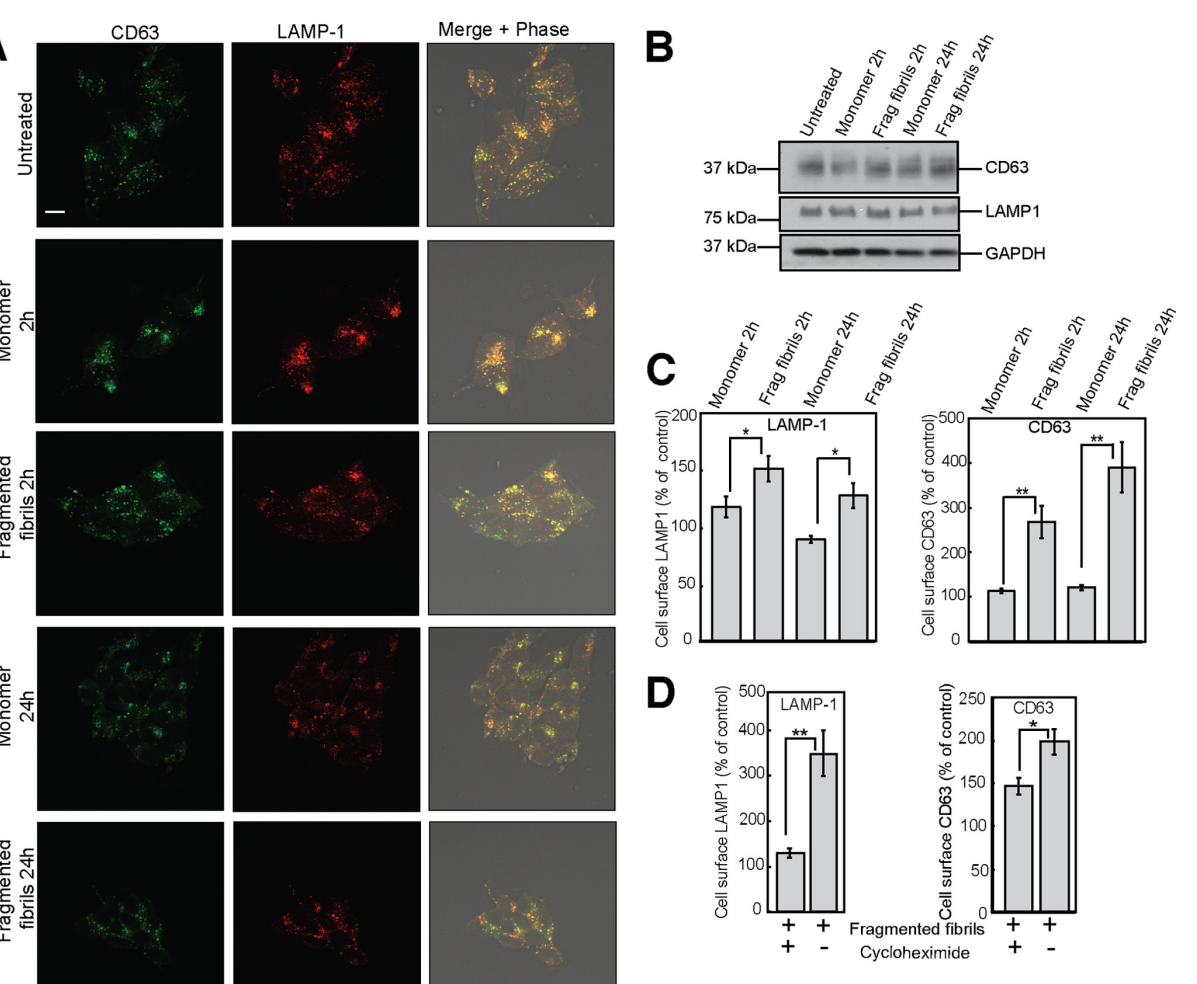
**Fragmented fibrils of β_2_m increase the cell surface expression of LAMP-1 and CD63.** SH-SY5Y cells were incubated with 1.2 μm (monomer-equivalent concentration) fragmented β_2_m fibrils or β_2_m monomers for 2 and 24 h. *A*, cells were fixed, permeabilized, and stained with LAMP1 and CD63 antibodies. Antibody staining was detected with fluorescently labeled secondary antibodies. Cell-associated fluorescence was visualized by confocal microscopy. In the *merged images*, images of antibody staining were combined with phase-contrast images. *Yellow* indicates colocalization of CD63 (*green*) and LAMP-1 (*red*). *B*, cell lysates were analyzed by immunoblotting with CD63-, LAMP-1-, and GAPDH-specific antibodies. *Scale bar* = 10 μm. *C*, cell surface expression of LAMP-1 and CD63 was quantified by flow cytometry and is expressed as a percentage of that of cells incubated for 0 h in the presence of β_2_m samples. *Error bars* indicate mean ± 1 S.E. over a total of nine replicates. *, *p* < 005; **, *p* < 0.01. *D*, SH-SY5Y cells were preincubated for 1 h in the presence or absence of cycloheximide and then incubated with fragmented β_2_m fibrils for 2 h. Cells were then stained with LAMP-1- and CD63-specific antibodies, and antibody fluorescence was quantified by flow cytometry.

To explore in more detail the impact of fragmented fibrils on lysosomes, we examined whether there was an alteration in the trafficking of the lysosomal membrane proteins LAMP-1 and CD63. LAMP-1 and CD63 exhibited a punctate distribution in untreated cells that was not altered substantially in cells that were incubated with either fragmented β_2_m fibrils or with β_2_m monomers (Fig. 8*A*). Immunoblotting of whole cell lysates also showed that levels of LAMP-1 and CD63 were comparable for cells incubated in the absence of β_2_m, with monomeric protein, or with fibrils (Fig. 8*B*). However, flow cytometry revealed that the incubation of cells with fragmented β_2_m fibrils resulted in increased cell surface expression of both LAMP-1 and CD63 in comparison with cells incubated with β_2_m monomers (Fig. 8*C*). These data indicate that fragmented β_2_m fibrils cause the redistribution of a subpopulation of LAMP-1 and CD63 to the plasma membrane. This could result from the trafficking of preexisting pools of these proteins to the plasma membrane, as would occur in lysosome exocytosis (54). Alternatively, an increased proportion of newly synthesized LAMP-1 and CD63 may traffic to, or be retained at, the plasma membrane (54, 55). Cells were therefore incubated with the protein synthesis inhibitor cycloheximide prior to the addition of fragmented β_2_m fibrils. This resulted in a reduction in the cell surface expression of LAMP-1 and CD63 (Fig. 8*D*), suggesting that a significant proportion of LAMP-1 and CD63 on the surface of cells incubated with the fibrils corresponds to newly synthesized protein.

##### Fragmented β_2_m Fibrils Inhibit Lysosomal Proteolysis

**FIGURE 9.**
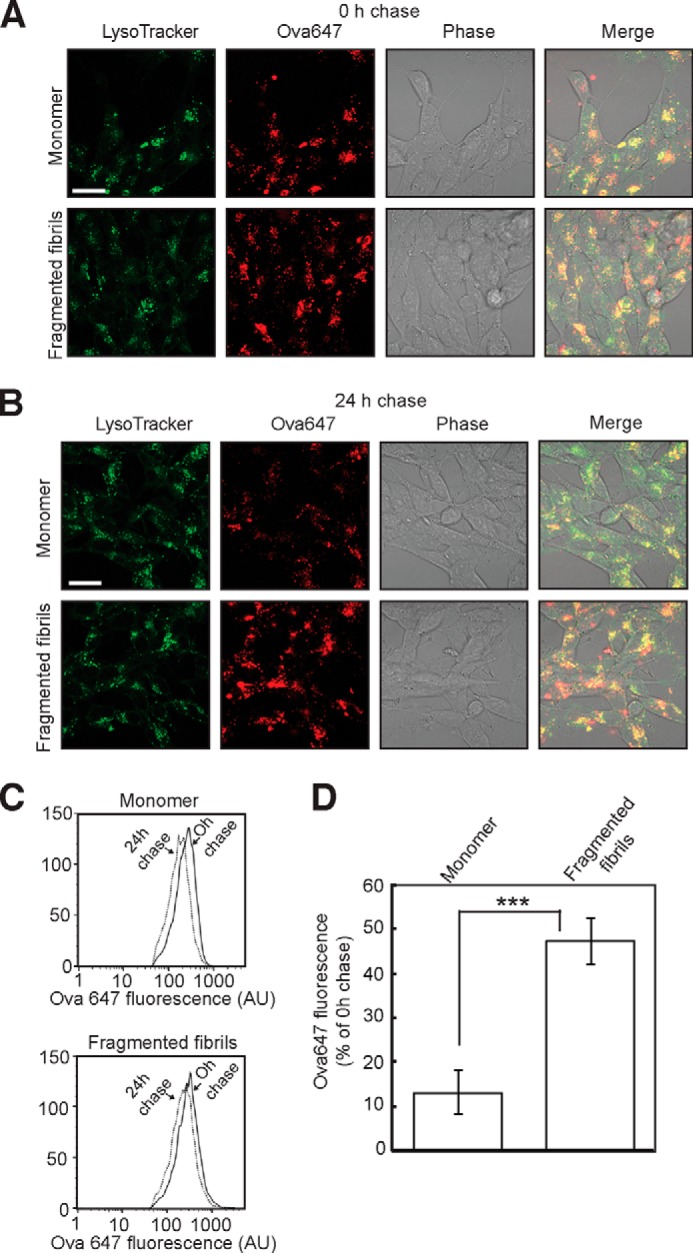
**Fragmented β_2_m fibrils inhibit the degradation of ovalbumin by lysosomes.** *A* and *B*, cells were incubated with either 1.2 μm (monomer-equivalent concentration) fragmented β_2_m fibrils or β_2_m monomers for 24 h. Cells were washed to remove non-cell-associated fibrils, pulsed with Ova-647 for 6 h, washed to remove non-cell-associated Ova-647, and either imaged immediately by live cell confocal microscopy (*A*) (*0 h chase*) or chased in the absence of Ova-647 for 24 h (*B*). Prior to imaging, cells were stained with LysoTracker Green. *Scale bar* = 10 μm. Cell-associated Ova-647 fluorescence was also quantified by flow cytometry. *C*, representative flow cytometry profiles. *AU*, arbitrary units. *D*, cell-associated Ova-647 fluorescence is expressed as the percentage of the fluorescence at 0 h chase remaining after 24 h chase. *Error bars* indicate mean ± 1 S.E. over nine replicates performed in three independent experiments. ***, *p* < 0.001.

Finally, to determine whether fragmented β_2_m fibrils disrupt the function of lysosomes, we assayed the cleavage of substrates for lysosomal hydrolases in SH-SY5Y cells. The capacity of lysosomes to degrade proteins was studied by measuring the proteolysis of ovalbumin, a protein that is endocytosed and degraded in lysosomes (56). Cells that had been preincubated with either monomeric β_2_m or fragmented fibrils were incubated with Ova-647, ovalbumin labeled with the fluorescent dye Alexa Fluor 647. Imaging experiments revealed that Ova-647 was internalized and sorted into punctate compartments that costained with LysoTracker Green (Fig. 9*A*). Cells were then washed to remove non-cell-associated Ova-647 and incubated for a further 24-h chase period prior to analysis. Cell-associated Ova-647 at the end of the chase period was visualized by confocal microscopy (Fig. 9*B*) and quantified by flow cytometry (Fig. 9, *C* and *D*). After the 24-h chase, cells preincubated with monomeric β_2_m had a pronounced reduction in cell associated Ova-647, <15% of that present at 0 h chase (Fig. 9, *B–D*). This was presumably due to the degradation of ovalbumin by lysosomal proteases (56). Cells preincubated with fragmented β_2_m fibrils, by contrast, had a significantly higher level of cell-associated Ova-647 after the 24-h chase, corresponding to ∼45% of that present at 0 h chase (Fig. 9, *B–D*). These data suggest, therefore, that amyloid fibrils impair the ability of SH-SY5Y cells to degrade endocytosed proteins.

**FIGURE 10.**
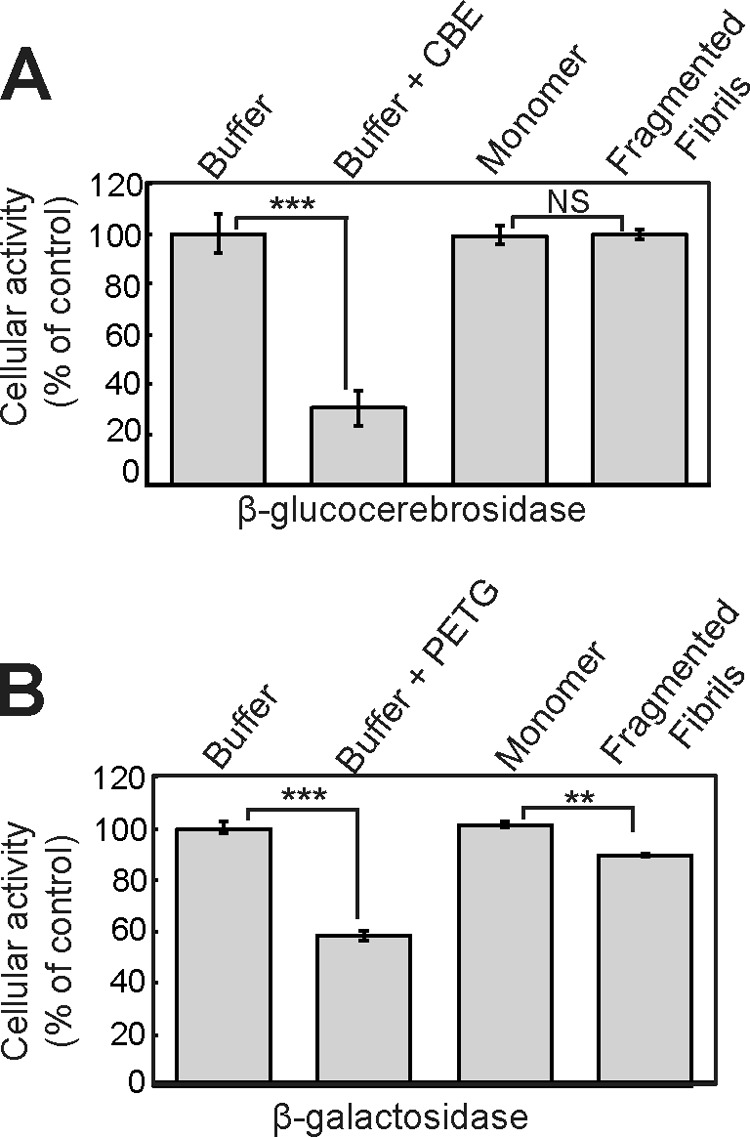
**Fragmented β_2_m fibrils have a limited effect on the cellular activities of β-glucocerebrosidase and β-galactosidase.** *A* and *B*, cells were incubated with either 1.2 μm (monomer-equivalent concentration) fragmented β_2_m fibrils or β_2_m monomers for 24 h. Cellular β-glucocerebrosidase (*A*) and β-galactosidase (*B*) activities were assayed using the fluorescent substrates 5-(pentafluorobenzoylamino)fluorescein di-β-d-glucopyranoside and C_12_FDG, respectively. As a control, cells were preincubated for 1 h with inhibitors of β-glucocerebrosidase (*CBE*) and β-galactosidase (phenylethyl β-d-thiogalactopyranoside (*PETG*)). Percent cellular activity relative to control cells incubated with the fibril growth buffer was plotted. *Error bars* indicate mean ± 1 S.E. over a total of six replicates performed in two independent experiments. *NS*, *p* ≥ 0.05; **, *p* < 0.01; ***, *p* < 0.001.

To determine whether the activity of other lysosomal hydrolases was affected by fragmented β_2_m fibrils, the cellular activities of the glycosidases β-glucocerebrosidase and β-galactosidase were assayed by flow cytometry using the substrates 5-(pentafluorobenzoylamino)fluorescein di-β-d-glucopyranoside and C_12_FDG, respectively (37, 38). The β-glucocerebrosidase inhibitor conduritol B epoxide, but not fragmented β_2_m fibrils, caused a significant reduction in the hydrolysis of 5-(pentafluorobenzoylamino)fluorescein di-β-d-glucopyranoside by SH-SY5Y cells (Fig. 10*A*). The hydrolysis of C_12_FDG was reduced by the β-galactosidase inhibitor phenylethyl β-d-thiogalactopyranoside, whereas incubation of cells with fragmented β_2_m fibrils resulted in a small reduction in substrate hydrolysis (Fig. 10*B*). Therefore, fragmented β_2_m fibrils inhibit the lysosomal degradation of ovalbumin but have a less pronounced effect on the cleavage of substrates by other lysosomal hydrolases.

## DISCUSSION

Despite decades of research, the culprit species and mechanisms of amyloid-associated cell dysfunction and cytotoxicity remain unclear. Many studies have focused on the early prefibrillar oligomeric intermediates of amyloid assembly, demonstrating that these species are cytotoxic (6–11). Here we demonstrate for the amyloidogenic protein β_2_m that reducing fibril length by fragmentation enables fibrils to be internalized more efficiently. For β_2_m fibrils, this results in the inhibition of MTT reduction and the perturbation of the endolysosomal pathway. Therefore, β_2_m amyloid fibrils behave as nanoparticles whose biological properties are dependent on their physical dimensions (57, 58).

Increased access of intracellular compartments to fragmented β_2_m fibrils via endocytosis rationalizes our previous observations of an inverse relationship between amyloid fibril length and the inhibition of MTT reduction (34). Correspondingly, the inhibition of fibril internalization with Dynasore rescued cellular MTT reduction. Other studies also suggest that the endocytosis of assemblies with an ordered β-sheet structure may be an important factor in amyloid disease, for example in the cytotoxicity of Ure2p amyloid-like protofibrils and Aβ_1–42_ oligomers (59–61); synaptic disruption induced by Aβ_1–42_ oligomers (62); intracellular aggregate formation by Aβ_1–40_, α-synuclein, polyglutamine sequences, and superoxide dismutase-1 (63–66); and for the activation of the inflammasome by Aβ_1–42_ fibrils (67). Analogous to our observations, inhibition of endocytosis can also protect cells against the deleterious effects of chemical fibrils (68). Conversely, our observations also explain why amyloid plaques are often seen as biologically inert (13) because such large assemblies would be inefficient in accessing intracellular compartments through endocytic pathways. Therefore, by affecting cellular uptake, particle size is likely to be a key determinant in the pathological responses of cells to aggregates formed in amyloid assembly reactions.

Despite inhibiting cellular MTT reduction, fragmented β_2_m fibrils did not cause cell death. Similar observations have been reported for microglia incubated with the amyloidogenic peptide Aβ_1–42_, in which MTT reduction was inhibited without any corresponding decrease in ATP levels or lactate dehydrogenase release (69). Therefore, in some instances, the inhibition of MTT reduction may correspond to a cellular response to amyloid that does not result in death. Notably, the incubation of SH-SY5Y cells with β_2_m fibrils coincided with the increased production of extracellular needle-like MTT formazan crystals. This effect has been observed for other amyloid sequences and has been attributed to an increase in the exocytosis of reduced MTT formazan and the resultant inhibition of MTT endocytosis (45–47, 69, 70), which may represent an alteration in intracellular trafficking pathways.

Our observation that fragmented β_2_m fibrils increased the cell surface expression of the lysosomal membrane proteins LAMP-1 and CD63, but not the total cellular content of these proteins, provides evidence that amyloid fibrils alter trafficking in the endolysosomal pathway. Because the increase in cell surface LAMP-1 and CD63 was dependent, in part, on new protein synthesis, fragmented β_2_m fibrils may divert an increased proportion of newly synthesized LAMP-1 and CD63 to the plasma membrane instead of these proteins being sorted directly from the Golgi to the endolysosomal pathway. Notably, loss of function of the AP-3 adaptor protein complex, which is involved in the sorting of proteins to lysosomes, also results in elevated cell surface expression of LAMP-1 and CD63 (71). Alternatively, fibrils may inhibit the endocytosis of a subset of LAMP-1 and CD63 molecules that traffic to lysosomes via the plasma membrane. The notion that trafficking may be altered in amyloid disorders is also supported by genetic studies of Parkinson disease, in which disease-associated mutations in VPS35, Rab7L1, and LRRK2 result in Golgi and endolysosomal trafficking defects (72, 73), whereas phosphatidylinositol-binding clathrin assembly protein, a protein with a role in endocytosis, is a modulatory factor in Alzheimer disease (74–76).

The inhibition of the degradation of ovalbumin by fragmented β_2_m fibrils provides further evidence for the perturbation of the endolysosomal pathway. The inhibition of the proteolysis of ovalbumin did not appear to result from damage to the lysosomal membrane by fragmented β_2_m fibrils. We cannot exclude the possibility that fibrils may cause transient increases in membrane permeability that were not detected in our experiments, although any substantive damage to the lysosome membrane would result in cell death (77), which was not observed in cells incubated with fragmented β_2_m fibrils. Moreover, LysoTracker Green staining also suggests that fibrils do not cause any pronounced increase in the pH of lysosomes. The accumulation of fibrils within lysosomes may instead overwhelm the proteolytic capacity of this organelle. This would explain why proteolysis of ovalbumin was reduced substantially, whereas there was a less pronounced effect on the cellular activity of the glycosidase β-galactosidase and no effect on β-glucocerebrosidase.

A reduction in the ability of lysosomes to degrade proteins would be predicted not only to impair the degradation of endocytosed soluble proteins, as shown here, but also that of membrane proteins such as receptors down-regulated from the plasma membrane (78). In addition, the degradation of proteins by autophagy is dependent on lysosomal proteolysis (79). The inhibition of lysosomal proteolysis by amyloid fibrils could be a key factor in amyloid disorders. Indeed, reduction of lysosomal proteolysis results in an Alzheimer-like axonal dystrophy (80), knockout of cathepsin D promotes Tau neurotoxicity (81), and an age-related reduction in the activity of cathepsin B and D is associated with the production of amyloidogenic fragments of amyloid precursor protein (82). Moreover, the loss of β-glucocerebrosidase activity impairs lysosome proteolysis, promoting α-synuclein accumulation and neurotoxicity (83).

Impaired lysosome function may also contribute to the pathology of dialysis-related amyloidosis, a disorder in which β_2_m fibrils form plaques in the osteoarticular tissues (32). Macrophages recruited to these amyloid plaques phagocytose β_2_m amyloid, which then accumulates within their lysosomes (33, 49, 84, 85). Our data predict that uptake of β_2_m fibrils would inhibit the degradative capacity of these cells. Furthermore, the disruption of lysosome function may be related to the inhibition by β_2_m fibrils of the resorption of a bone-like matrix by osteoclasts (33), a cell type that utilizes lysosome-like organelles to remodel bone (86).

In summary, our observations provide further evidence that the lysosome represents a key cellular target in amyloid disease (87, 88) and that this organelle is readily accessible to nanoscale amyloid fibrils produced early during amyloid assembly reactions or by the fragmentation of existing fibrils. Inhibiting fibril fragmentation and preventing fibril access to intracellular compartments may protect cells and provide effective strategies toward combating amyloid disease.

## Footnotes

5 The abbreviations used are:

β_2_m: β_2_-microglobulin
MTT: 3-(4,5-dimethylthiazol-2-yl)-2,5-diphenyltetrazolium bromide
TMR: tetramethylrhodamine
C_12_FDG: 5-dodecanoylaminofluorescein di-β-d-galactopyranoside.
